# Postnatal changes in the development of rat submandibular glands in offspring of diabetic mothers: Biochemical, histological and ultrastructural study

**DOI:** 10.1371/journal.pone.0205372

**Published:** 2018-10-10

**Authors:** Abir El Sadik, Enas Mohamed, Ahmed El Zainy

**Affiliations:** 1 Department of Anatomy and Embryology, Faculty of Medicine, Cairo University, Cairo, Egypt; 2 Qassim University, Qassim, KSA; National Institute of Dental and Craniofacial Research, UNITED STATES

## Abstract

Development and maturation of submandibular salivary glands are influenced by intrauterine diabetic environment. Several studies investigated the effects of diabetes on the salivary glands. However, the effects of maternal diabetes on the submandibular glands of the offspring was not properly examined. Therefore, the present study was designed to describe the changes in the development of the submandibular glands of the offspring of diabetic mothers. The submandibular glands of the offspring of Streptozotocin (STZ)-induced diabetic female rats were examined at two and four weeks after birth. Detection of mRNA demonstrated that maternal diabetes affects the level of different indicators. The reduction of expression of epidermal growth factor (EGF); a protein mitogen, cytokeratin 5 (CK5); an epithelial cell progenitor, CK7 and aquaporin 5 (AQP5); differentiation markers and B cell lymphoma 2 (Bcl2); an antiapoptotic marker were found. Increase in Bcl2-associated X protein (Bax); an apoptotic marker was detected. These changes indicate their effects on saliva secretion, glands tumorigenesis, growth of normal oral flora and oral microbes, with decreased protein synthesis and production of xerostomia and dental caries. Loss of normal glandular architecture, significant increase in fibrosis, by the detection of collagen fibers, and stagnation of secretory granules were found with atrophic changes in the acinar cells. Marked defect of polysaccharides in the acinar cells, denoting functional changes, was manifested by significant reduction of the intensity of periodic acid-Schiff (PAS) reaction. The positive immunoreactivity of caspase-3, denoting cellular apoptosis, and minimal reaction of alpha-smooth muscle actin (α SMA) and proliferating cell nuclear antigen **(**PCNA) were evident in the offspring of diabetic mothers. We conclude that maternal diabetes produces degenerative effects in the structure and function of the submandibular salivary glands of the offspring, reflecting possible influences on their secretory activity affecting oral and digestive health.

## Introduction

Development of human salivary glands starts from the fourth week of fetal life and continues after birth [1]. Therefore, maturation of the submandibular salivary glands is influenced by intrauterine and postnatal disturbances. The glands develop in a complex and coordinated dynamic process beginning from the proliferation of solid cord of epithelial cells, then branching, formation of lobules, cords canalization and cytodifferentiation [2]. The submandibular glands produce 65% of the total saliva secreted from all salivary glands. Movement of the saliva is necessary for the removal of bacteria, pH buffering and oral health generally. The high shear rates during eating and swallowing maintain a constant flow of saliva from the ductal openings in the mouth to swallowing into the back of the throat [3]. Secretion of saliva increases above the resting level by taste and mastication and to a lesser degree by olfaction [4]. Carpenter [3] concluded that the principle stimulus for the flow of saliva are chewing and taste. In addition, the antibacterial, antiviral and antifungal components of the saliva help to maintain the normal oral flora [5]. Benn and Thomson [6] reported that submandibular glands secrete viscous saliva composed of 99% water. Other components include sodium, potassium, calcium, magnesium, bicarbonate, phosphates, immunoglobulins, proteins, glycoproteins, mucins, urea, ammonia and sulfated cystatins. Moreover, submandibular salivary secretion contains neuronal and epidermal growth factors which promote lubrication and protection of gums and oral mucosa. Most calcium in saliva is bound to statherin or to other phosphor-containing proteins that prevents excessive precipitation of calcium on the teeth [7].

Alteration in the structure and integrity of the glands affects their function and activity and produces changes in the saliva flow and composition, disturbing oral health [8, 9]. Amylase enzymes secreted in saliva play an important role in starch digestion, contributing significantly to overall metabolic status. Salivary amylase breaks down starch into simpler sugar molecules that could be absorbed into the bloodstream. Therefore, altered gland morphology affects blood glucose levels which have a direct impact on the maintenance of good health [10]. Moreover, Carpenter [3] concluded that salivary amylase is a specific enzyme that converts maltose to glucose and many non-soluble complex polysaccharides into smaller soluble units. Although, salivary amylase plays a less important role in digestion of starch compared to pancreatic amylase. It may be more important in the clearance of food after chewing. This results in decrease in the existence of substrates need for growth of microbes [11].

The growth and development of the fetus during pregnancy is determined by multiple environmental factors. One of the most important factors is nutrition. Organogenesis of the fetus is markedly affected by any changes in the plasma nutrient levels of the maternal blood and consequently the fetal plasma composition [12]. Clinical studies suggested that human fetuses of diabetic mothers are exposed to over nutrition and fuel mediated teratogenicity that could have long term impact on the metabolic functions of the offspring [13]. Uterine diabetic environment was demonstrated to produce higher rates of obesity, glucose intolerance and type 2 diabetes mellitus in the offspring [14].

Several studies have reported the influence of experimental diabetes on rat submandibular salivary glands [15, 16]. Fedriko et al. [17] induced experimental diabetes, after single intraperitoneal injection of streptozotocin, which produced disruption of the function of the submandibular glands attributed to increase in calcium load. In addition to its mobilizing ability of cholinergic receptors with subsequent reduction in the Ca^2+^-ATPases activity and endoplasmic reticulum Ca^2+^content. Induction of diabetes with alloxan can cause certain functional and morphological changes in the submandibular glands affecting their secretions. This could be attributed to an oxidative stress causing degeneration and apoptosis in submandibular stromal cells [18].

However, the study of the effects of maternal diabetes on the submandibular salivary glands of the offspring was not properly investigated. Therefore, the current study was performed to determine the biochemical, histological, immunohistochemical and ultrastructural postnatal changes in the development of the submandibular salivary glands of the offspring of diabetic mothers.

## Material and methods

### Animals

A total of twenty adult naïve female Sprague Dawley albino rats weighting, 200–250 g each and 4–5 months old, were used in the present study. The female rats were divided with balanced weight into two groups; one control group and one diabetic group. Each group is subdivided into two subgroups; five females per cage. One adult male albino rat was added, for breeding, to each subgroup of females; each subgroup was formed of five females and one male to copulate. They were left for two nights, then fertilization was confirmed by vaginal smear examination every morning to every female for one week. The presence of vaginal plug was designated as day zero of gestation. After confirming pregnancy of all females, the male rats were removed. The rats were locally bred at the animal house at Faculty of Medicine, Cairo University, Egypt. They were housed at room temperature, had access to food and water ad libitum. The animals were given two weeks’ acclimatization period before starting the experiment. They were treated in accordance with the international guidelines for the care and use of laboratory animals and the experiment protocol was approved by the Ethics Committee, Faculty of Medicine, Cairo University.

### Induction of diabetes mellitus

Rats were fasted for 12 hours before diabetes was induced using Streptozotocin (STZ) (Sigma, Aldrich, MO, USA). STZ was freshly dissolved in 0.05 M citrate buffer (pH 4.5). The diabetic group was injected with STZ at a dose of 60 mg/kg of the body weight intravenously. STZ induces diabetes within three days by destroying the beta cells. Therefore, diabetes was induced to the female rats three days before breeding with the males. The control group was injected with 0.05 M citrate buffer intravenously [19].

### Serum glucose levels and body weight

The induced diabetic state was assessed by monitoring serum glucose levels. The rats were fasted for eight hours before measurements. Blood samples were collected by retro-orbital plexus technique using capillary glass tubes. The collected blood samples were analyzed for serum glucose levels by the glucose oxidase peroxidase (GOP) method [20]. Rats with blood glucose levels exceeding 200 mg/dl at 24 h after treatment were considered diabetic. The blood glucose level of only two rats of one diabetic subgroup exceeded 400 mg/dl at the 7^th^ day of pregnancy. The blood glucose level, of these two rats, was monitored daily and they were administered 10 units of insulin (Mixtard 30, Novo Nordisk, Copenhagen, Denmark) subcutaneously daily till the end of the experiment [21]. The body weight and serum glucose level of diabetic and nondiabetic mothers were measured before pregnancy and at the 7^th^, 14^th^ and 21^st^ days of pregnancy in addition to the serum glucose level of the offspring at the second and fourth weeks after labor.

### Experimental design

Only male offspring were used in this study to avoid insulin resistance which is more common in females. Vital et al. [22] found significant differences in insulin sensitivity between sexes, female rats had lowered insulin sensitivity than males. Ten male offspring were randomly collected from the five mothers in each subgroup; two offspring from each mother, except one diabetic subgroup; ten rats were randomly collected from only four mothers who successfully conceived (four offspring from two mothers and six offspring from the other two mothers). All litters born survived to the study age. The rats were sacrificed, by overdose of anesthesia; 40 mg/kg body weight pentobarbital intraperitoneally, at two weeks and four weeks after birth forming the following groups:

**Group I (2w control):** Control group of 2 weeks old offspring of nondiabetic mothers.

**Group II (2w diabetic)**: 2 weeks old offspring of diabetic mothers.

**Group III (4w control):** Control group of 4 weeks old offspring of nondiabetic mothers.

**Group IV (4w diabetic):** 4 weeks old offspring of diabetic mothers.

The submandibular glands were dissected from the rats.

### Detection of studied genes by quantitative real time polymerase chain reaction (QRT-PCR)

**Fig 1:**
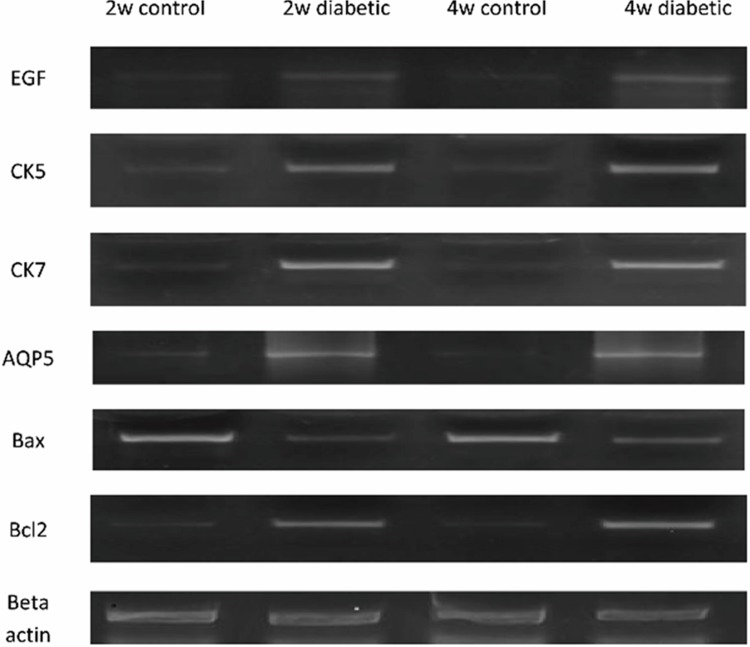
QRT-PCR products of EGF, CK5, CK&, AQP5, Bax, Bcl2 and beta actin gene expression in different studied groups.

The submandibular glands were preserved at -80°C in phosphate buffer saline (PBS) pH 7.4. Total RNA was extracted from submandibular gland tissue from the offspring homogenate using RNeasy purification reagent (Qiagen, Valencia, CA) per the manufacturer’s instructions. cDNA was generated from 5 μg of total RNA extracted with 1 μl (20 pmol) antisense primer and 0.8 μl superscript AMV reverse transcriptase for 60 min at 37°C. The relative abundance of mRNA species was assessed on an ABI prism 7500 sequence detector system (Applied Biosystems, Foster City, CA). PCR primers were designed with Gene Runner Software (Hasting Software, Inc., Hasting, NY) from RNA sequences from GenBank (Fig 1 and Table 1). All primer sets had a calculated annealing temperature of 60°C. Quantitative RT-PCR was performed in duplicate in a 25-μl reaction volume consisting of 2X SYBR Green PCR Master Mix (Applied Biosystems), 900 nM of each primer and 2 μl of cDNA. Amplification conditions were 2 min at 50°, 10 min at 95° and 40 cycles of denaturation for 15 s and annealing/extension at 60°C for 10 min. Data from real-time assays were calculated using the v1·7 Sequence Detection Software from PE Biosystems (Foster City, CA). Relative expression of the studied genes was calculated using the comparative threshold cycle method. All values were normalized to the beta-actin gene. The relative quantification was calculated by the expression 2^-ΔΔCt^ and reported as fold change of the background level detected in 2w control and 4w control groups [23].

**Table 1:** Primer sequences used for the QRT-PCR.

	Primer sequences
**EGF**	Forward primer: 5′ AGCCCGGTTTTGTTAAGTG3′ Reverse primer: 5′ AGTATCGCCTTCCAGTGTC 3′
**CK5**	Forward primer: 5′-TGGTCTCCCGTGCCGCAGTTCTAT -3′, Reverse primer: 5′-ATTTGGGATTGG CTGTGGG-3′,
**CK7**	Forward primer: ‘5TGCCAAGTTAGAGTCCAGCA -3′ Reverse primer: 5′-TTGATGGAATAGGCCCTGAG-3′
**AQP5**	Forward primer: 5′-GGGATCTACTTCACTGGCTG-3′ Reverse primer: 5′-GGTCTTGGTGTCAGGGAACA-3′
**Bax**	Forward primer: 5′-GTTGCCCTCTTCTACTTTG -3′ Reverse primer: 5′-AGCCACCCTGGTCTTG-3′
**Bcl2**	Forward primer: 5′ CGGGAGAACAGGGTATGA 3′ Reverse primer: 5′ CAGGCTGGAAGGAGAAGAT 3′
**β-actin**	Forward primer:’5CCAGGCTGGATTGCAGTT’3 Reverse primer: ‘5GATCACGAGGTCAGGAGATG’3

EFG: epidermal growth factor, CK5: cytokeratin5, CK7: cytokeratin7, AQP5: aquaporin5, Bax: Bcl2-associated X protein, Bcl2: B cell lymphoma2

### Light Microscopic Examination

The submandibular glands were fixed in 10% formalin solution, processed, embedded to obtain paraffin blocks, and cut at 5 μm thickness sections. The sections were deparaffinized in xylol solution then rehydrated in 100%, 95%, 70% alcohol and washed in distilled water and subjected to the following stains:

#### Hematoxylin and eosin (H & E) staining

The sections were stained with hematoxylin for ten minutes. The nuclei and the basophilic structures of the cytoplasm were stained with blue color. Excess stain was removed by decolorization in one percent hydrochloric acid in 70% alcohol for five seconds. Then the sections were stained in one percent aqueous eosin for three minutes to stain the acidophilic structures of the cytoplasm with red color. The sections were dehydrated in alcohol (70% for one minute, 90% for three minutes then 100% for other three minutes) then cleared from alcohol by xylene. The sections were mounted in Canada balsam by removing quickly the slide from xylol with its face downwards and putting it on the cover slip [24].

#### Masson’s trichrome staining

Masson’s trichrome stain was performed to assess the increase in amount of collagen fibers denoting fibrosis. The sections were stained in Weigert’s iron hematoxylin solution for 10 min, rinsed in running tap water for 10 minutes and washed in distilled water. Then they were stained in Biebrich scarlet-acid fuchsin solution for 15 minutes, washed in distilled water and differentiated in phosphomolybdic-phosphotungestic acid solution for 10 minutes. Then the sections were transferred to aniline blue solution for ten minutes, rinsed shortly in distilled water and differentiated in 1% acetic acid solution for five minutes then rewashed in distilled water. The sections were dehydrated very quickly through absolute ethyl alcohol, cleared in xylene and finally mounted in Canada balsam. Collagen fibers appeared blue [25].

#### Periodic acid-Schiff (PAS) staining

Periodic acid-Schiff (PAS) staining was performed to evaluate the presence of polysaccharides in the acinar cells, that indicates functional changes. One-gram periodic acid was dissolved in 200 ml distilled water. One gm basic fuchsin was dissolved in 200 ml of boiling distilled water to produce Schiff’s reagent. The solution was allowed to cool to 50°C and two grams potassium metabisulfite were added while mixing. Two ml concentrated hydrochloric acid were added after allowing the solution to cool to room temperature. Two grams of activated charcoal were added and left overnight in the dark at room temperature. The sections were treated with periodic acid for five minutes then washed with distilled water then covered with Schiff’s solution for 15 minutes. After washing with running tap water, the nuclei were stained with Harri’s hematoxylin, differentiated in acid-alcohol and blued as usual. The sections then washed in water, rinsed in absolute alcohol, cleared in xylene and mounted as desired. The polysaccharides were homogenously stained in the cells of the acini [26].

### Immunohistochemistry

Sections were cut at 5 μm thickness and collected on poly-L-lysine coated slides, then deparaffinized in two changes of xylene, 10 minutes each, and hydrated through graded washes of ethanol in water, and finally rinsed in pure water. Nonspecific endogenous peroxidase activity was blocked by treatment with 0.9% hydrogen peroxide in absolute methanol for 10 min. Antigen retrieval was performed by heating the sections in 10 mM sodium citrate buffer, in a water bath at 100°C for 30 minutes. Sections were rinsed twice in PBS Tween 20 for 2 minutes and blocked with 5% normal mice serum for 30 minutes at room temperature. They were incubated with the following primary antibodies for 30 minutes:

1. Caspase-3 antibody, primary antisera diluted in antibody diluents (1:500) (TA-125-UD, Lab vision, Goteborg, Sweden). The peroxidase activity was demonstrated by AEC (3-amino-9-ethyl carbazole) substrate kit (TA- 004HAC, Lab vision, Goteborg, Sweden). Positive reaction in the form of brown discoloration of the cytoplasm was shown in the apoptotic cells while the normal cells showed negative reaction with absence of the brown discoloration.
2. α Smooth Muscle Actin (α SMA), mouse monoclonal antibody (AB-7817, abcam medical, Cambridge, MA, USA) was diluted 1:100 in 0.05 mol/L Tris-HCl, pH 7.6 containing stabilizing protein and 0.015 mol/L sodium azide. Histostain SP kit containing serum blocking solution, a biotinylated secondary antibody, a horseradish peroxidase streptavidin and a substrate chromogen mixture was purchased (95–9643, LAB-SA system, Zymed Laboratories Inc, San Francisco, CA, USA). Positive reaction appeared in the form of brown cytoplasmic deposits in the myoepithelial cells indicating their degree of proliferation.
3. Proliferating cell nuclear antigen (PCNA), a cofactor of DNA polymerase delta involved in DNA replication, was detected by rabbit polyclonal IgG (SC-7907, 200 μg/ml, dilution 1:50, Santa Cruz Biotechnology, USA). Positive reaction appeared in the form of brown colored nuclei in the proliferating cells.

### Electron Microscopic Examination

Specimens from the submandibular glands were cut into small slices, fixed in 4% glutaraldehyde then washed in phosphate buffer and post fixed in 1% osmium tetraoxide. Fixation was followed by dehydration in graded ethanol series (30%, 50%, 70%, 90%) for five minutes each at room temperature. Then dehydrated in 100% ethanol for ten minutes twice. The specimens were then immersed in a mixture of equal volume of ethanol (100%) and acetone (100%) for 15 minutes, then in 100% acetone for another 15 minutes twice. The specimens were embedded in equal volumes of epon and acetone for one hour, to facilitate their infiltration with epoxy resin. Semithin sections (1μm) were stained with toludine blue. The plastic-embedded tissue was sectioned with ultramicrotomes using diamond knives into ultrathin sections (50 nm) then picked up from the surface of a fluid-filled trough onto plastic-coated copper mesh grids. The grids were stained by a double staining technique of uranyl acetate, followed by lead citrate solution. These sections were examined and photographed using a Jeol, 100 CX II transmission electron microscope (Jeol, Tokyo, Japan). The grids were inserted in the specimen holder. At first low magnification was used (x1000) to locate the specimen, then, higher magnification was used to identify the cells and evaluate the findings using qualitative criterion that examined the morphological changes in the cellular organelles which were reflected on their secretory functions. Images were captured by CCD camera model AMT [27].

### Histomorphometric measurements

The measurements were taken by an independent observer, who was unaware of the experimental design. They were obtained in ten nonoverlapping fields per specimen at a magnification of 400 by using Leica LAS, V3.8 image analyser computer system (Switzerland). The image analyzer was first calibrated automatically to convert the measurement units (pixels) produced by the image analyzer program into actual micrometer units. One section for each animal in each group was examined under the light microscope; in each section ten non-overlapping fields were randomly chosen with fixed field area 4 x 10^4^ μm^2^. The area percent of collagen fibers in Masson's trichrome stained sections, polysaccharides in PAS stained sections and positive immune reaction for caspase-3, α SMA and PCNA stained sections were measured. The areas of the positive reaction or staining were highlighted for each image using MIA software by manually selecting the pixel values of each using the color cube-based tool in the count/size application. The area percent was selected in the program and generated automatically for each image [16].

### Statistical analysis

The data obtained for all groups were expressed as mean and standard deviation (± SD) and subjected to statistical analysis using “SPSS 22” software. The data were subjected to analysis of variance using independent t-test. Results were considered significant when p value was ≤0.05 [28].

## Results

### Serum glucose levels and body weight

**Fig 2:**
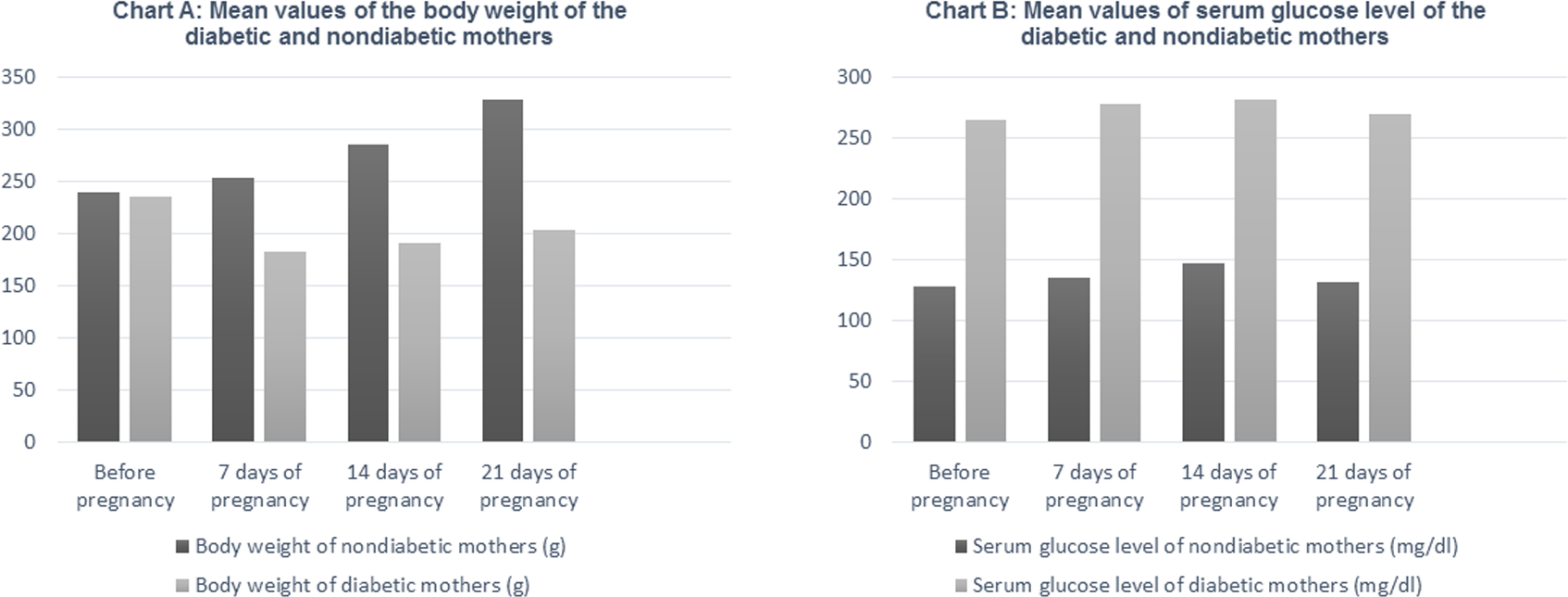
Chart A. Mean values of the body weight of the diabetic and nondiabetic mothers Chart B. Mean values of serum glucose level of the diabetic and nondiabetic mothers.

Significant decrease, in the body weights of all diabetic mothers, was found at the 7^th^, 14^th^ and 21^st^ days of pregnancy, compared with those of all nondiabetic mothers. However, the variation in body weights before pregnancy were nonsignificant. Significant increase in the serum glucose levels of all diabetic mothers, detected before pregnancy and at the 7^th^, 14^th^ and 21^st^ days of pregnancy, was found compared with those of nondiabetic mothers (Table 2 and Fig 2). On the other hand, no significant variation in the serum glucose levels of the offspring of all groups was found after labor (Table 3).

**Table 2:** Mean values ± standard deviation and significance of the body weight and serum glucose level of the diabetic and nondiabetic mothers before pregnancy and at the 7^th^, 14^th^ and 21st days of pregnancy and percent of increase or decrease.

	Before pregnancy	7 days of pregnancy	14 days of pregnancy	21 days of pregnancy
Body weight of nondiabetic mothers (g) and percent of weight increase or decrease	239 ± 16.08	253 ± 21.3 5.86%	285 ± 22.07 19.25%	328 ± 19.21 37.24%
Body weight of diabetic mothers (g) and percent of weight increase or decrease	235 ± 15.18	183 ± 13.4* -22.13%	191 ± 11.63* -18.72%	204 ± 16.2* -13.19%
Serum glucose level of nondiabetic mothers (mg/dl) and percent of level increase or decrease	128 ± 25.07	135 ± 21.19 5.47%	147 ± 22.6 14.84%	132 ± 20.11 3.13%
Serum glucose level of diabetic mothers (mg/dl) and percent of level increase or decrease	265 ± 22.03*	278 ± 24.08* 4.91%	281 ± 19.13* 6.04%	269 ± 21.07* 1.51%

*P value ≤ .05

**Table 3:** Mean values ± standard deviation and significance of the serum glucose level of the offspring.

	2w control	2w diabetic	4w control	4w diabetic
Serum glucose level (mg/dl)	133 ± 21.16	145 ± 18.02	137 ± 24.15	135 ± 15.61

### QRT-PCR for EGF, CK5, CK7, AQP5, Bax and Bcl2 gene expression

**Fig 3:**
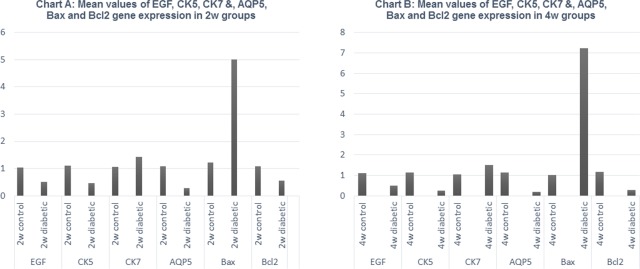
Chart A. Mean values of EGF, CK5, CK7&, AQP5, Bax and Bcl2 gene expression in 2w control and 2w diabetic groups Chart B. Mean values of EGF, CK5, CK7&, AQP5, Bax and Bcl2 gene expression in 4w control and 4w diabetic groups.

Detection of mRNA was performed demonstrating that maternal diabetes significantly reduced the expression of epidermal growth factor (EGF) which is a protein mitogen [29], cytokeratin 5 (CK5) which is an epithelial cell progenitor [30], aquaporin 5 (AQP5) which is an acinar [31] differentiation markers and B cell lymphoma 2 (Bcl2) which is an antiapoptotic marker and significantly increased Bcl2-associated X protein (Bax) which is an apoptotic marker [32] in 2w diabetic group compared with 2w control group (Table 4 and Fig 3) and in 4w diabetic group compared with 4w control group (Table 5 and Fig 3) demonstrating the degenerative effects of maternal diabetes on the development, growth and function of the submandibular salivary glands of the offspring. However, CK7; the ductal differentiation marker [33] didn’t show significant variation between the groups.

**Table 4:** Mean values, standard deviation and significance of QRT-PCR products of EGF, CK5, CK7&, AQP5, Bax and Bcl2 gene expression in the 2w control and 2w diabetic groups.

	Case	Mean	Std. Deviation
EGF	2w control	1.03	0.03
	2w diabetic	0.52*	0.23
CK5	2w control	1.10	0.07
	2w diabetic	0.46*	0.27
CK7	2w control	1.07	0.03
	2w diabetic	1.43	0.58
AQP5	2w control	1.08	0.10
	2w diabetic	0.28*	0.09
Bax	2w control	1.22	0.16
	2w diabetic	5.02*	1.10
Bcl2	2w control	1.09	0.09
	2w diabetic	0.55*	0.05

*P value ≤ .05

**Table 5:** Mean values, standard deviation and significance of QRT-PCR products of EGF, CK5, CK7&, AQP5, Bax and Bcl2 gene expression in the 4w control and 4w diabetic groups.

	Case	Mean	Std. Deviation
EGF	4w control	1.12	0.15
	4w diabetic	0.48*	0.58
CK5	4w control	1.15	0.18
	4w diabetic	0.24*	0.22
CK7	4w control	1.06	0.06
	4w diabetic	1.51	0.76
AQP5	4w control	1.15	0.08
	4w diabetic	0.18*	0.09
Bax	4w control	1.01	0.02
	4w diabetic	7.24*	0.63
Bcl2	4w control	1.16	0.12
	4w diabetic	0.28*	0.09

*P value ≤ .05

### Light Microscopic Examination

**Fig 4:**
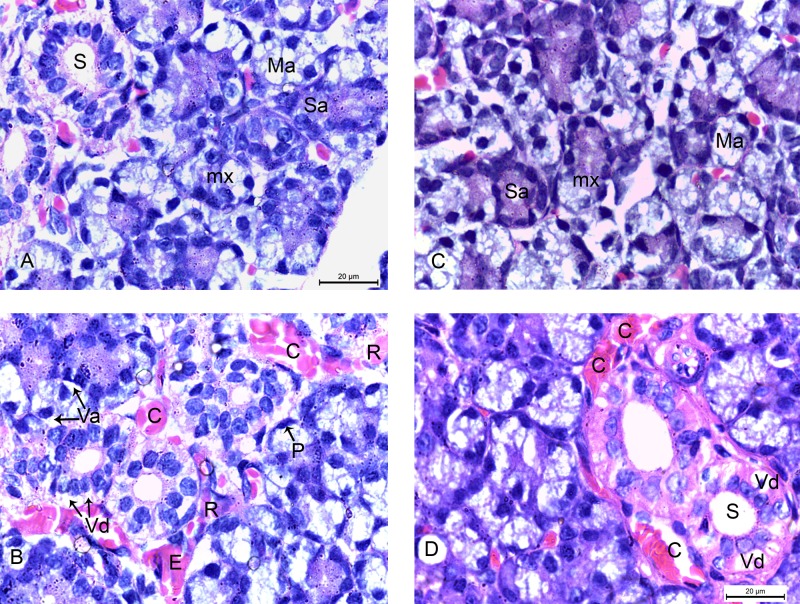
Photomicrographs of submandibular glands: A) (2w control group) showing normal serous (Sa), mucous (Ma) and mixed acini (mx) with striated ducts (S). B) (2w diabetic group) showing degeneration of the acini in the form of vacuolation of their cells with displacement of their nuclei (Va), vacuolation of the ductal cells (Vd) and pyknosis of their nuclei (P). Exudation (E) around the degenerated duct and congested capillaries (C) with extravasation of RBCs (R) are seen. C) (4w control group) showing normal serous (Sa), mucous (Ma) and mixed acini (mx). D) (4w diabetic group) showing shrinkage of the striated duct (S) with variable degree of cytoplasmic vacuolation (Vd). Dilated congested capillaries (C) are seen around the striated ducts. (H&E x 1000).

Histological examination, with haematoxylin and eosin stain, of the submandibular gland of 2w control group, presented the normal architecture of the glandular tissue. The specimens showed differentiated serous, mucous and mixed acini together with multiple striated ducts. The serous acini were lined with pyramidal cells with large basal nuclei and basophilic cytoplasm. The striated ducts were lined with columnar cells with acidophilic cytoplasm (Fig 4A). The submandibular glands of 2w diabetic group showed degenerative changes of the acini presented by vacuolation of their cells with displacement of their nuclei. The striated ducts appeared atrophic with many vacuoles around their cells and pyknosis of their nuclei. Exudation appeared around the degenerated ducts and the congested capillaries with extravasation of red blood cells (RBCs) among the glandular tissue (Fig 4B). The submandibular gland of 4w control group showed complete differentiation of the glandular acini (Fig 4C). The specimens of 4w diabetic group presented more degenerative changes especially in the striated ducts. The ducts showed shrinkage in their cells with variable degree of vacuolation in their cytoplasm. Congestion and dilatation of the capillaries were seen around the ducts (Fig 4D). All these morphological changes were found in all the specimens of the rats of 2w diabetic and 4w diabetic groups revealing the histopathological changes in the submandibular glands.

**Fig 5:**
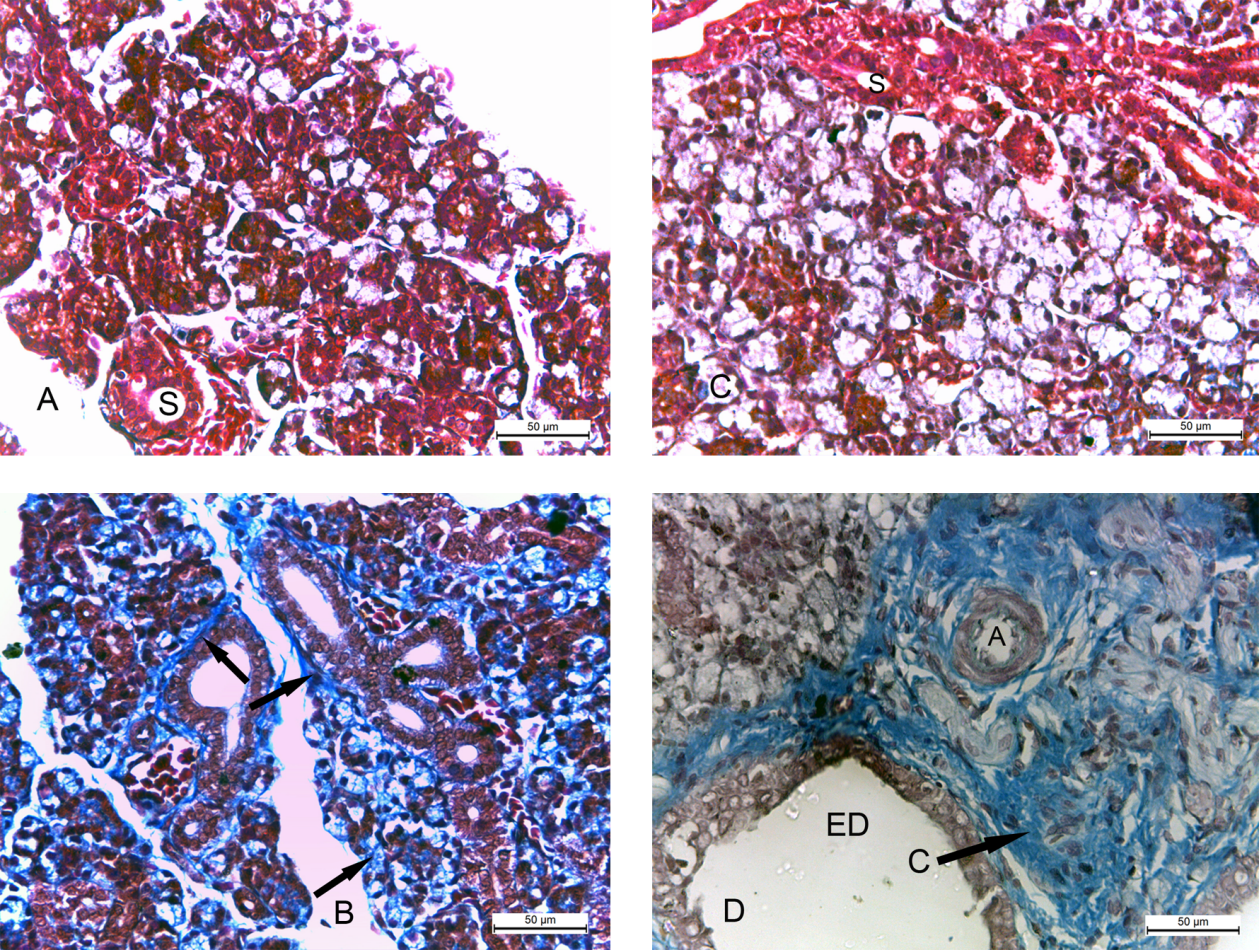
Photomicrographs of submandibular glands: A) (2w control group) showing absence of collagen fibers around the acini and the striated ducts (S). B) (2w diabetic group) showing large amount of collagen fibers (arrows) around the ducts and between the acini. C) (4w control group) showing absence of collagen fibers in the glandular tissue and around the striated ducts (S). D) (4w diabetic group) showing massive amount of collagen fibers (C) around the excretory duct (ED) and the thickened arteriole (A). Note the complete loss of acinar architecture. (Masson's trichrome x 400).

The submandibular glands of 2w control group stained with Masson’s trichrome stain showed absence of collagen fibers around the acini and the striated ducts (Fig 5A). The specimens of 2w diabetic group showed fibrosis of the glandular tissue in the form of large amount of collagen fibers surrounding the striated ducts and in between the acini (Fig 5B). The specimens of 4w control group showed absence of collagen fibers around the acini and the striated ducts (Fig 5C). The glands of 4w diabetic group showed massive amount of collagen fibers around the excretory ducts and thickened arterioles. Complete loss of acinar architecture was detected around the fibrosed area (Fig 5D).

**Fig 6:**
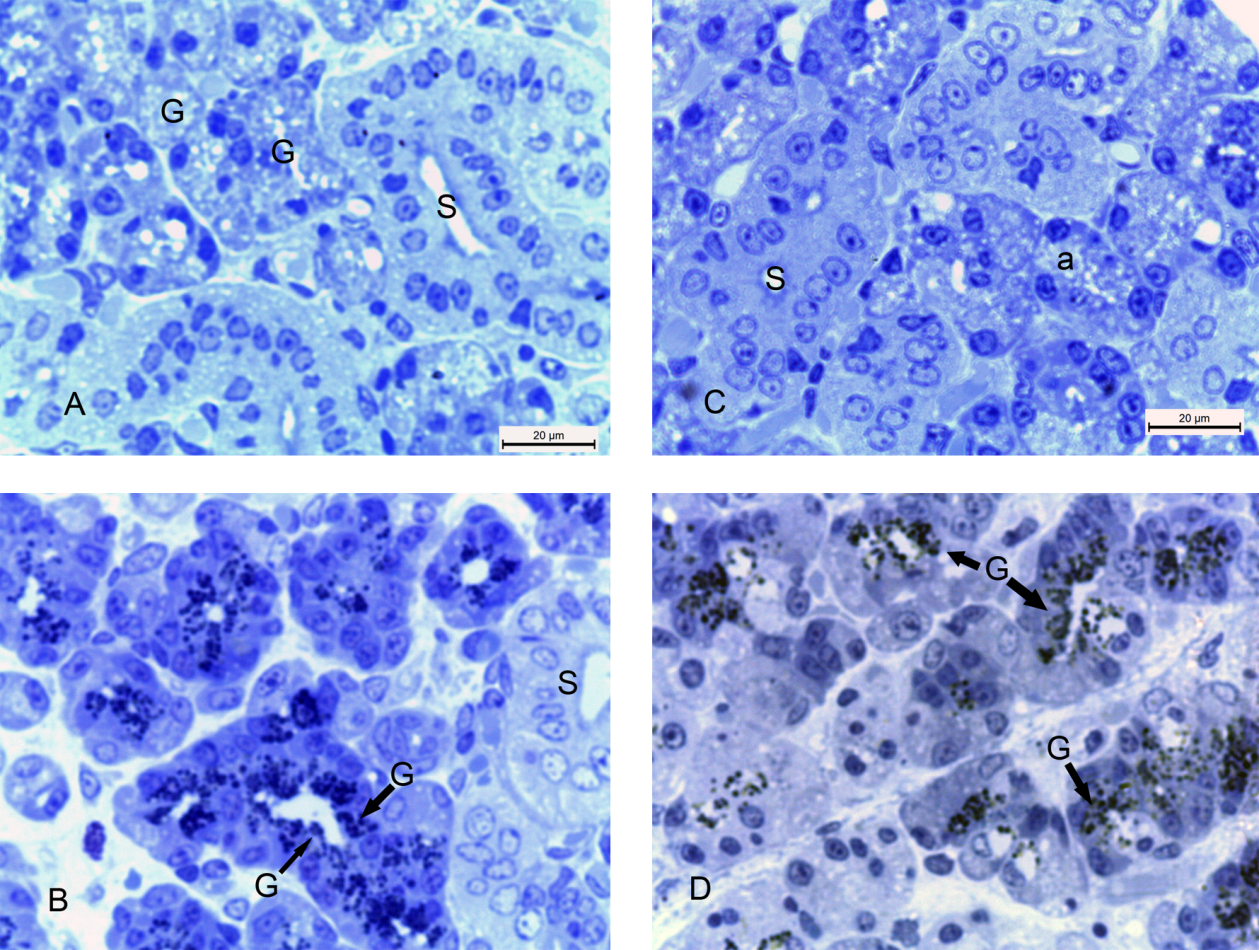
Photomicrographs of submandibular glands: A) (2w control group) showing striated ducts (S) and secretory granules (G) in the lumen and the cytoplasm of the acinar cells. B) (2w diabetic group) showing striated ducts (S) and large amount of stagnant secretory granules (G) C) (4w control group) showing normal striated ducts (S) and acini (a). D) (4w diabetic group) showing large amount of stagnant secretory granules (G) in some cells and the lumen of the acini. (Toludine blue x 1000).

Semithin sections of 2w control group, stained with toludine blue, showed secretory granules in the lumen and the cytoplasm of the acinar cells (Fig 6A). 2w diabetic group showed large amount of stagnant secretory granules in some cells (Fig 6B) compared with 2w control group. Accumulation of the secretion of the acini in their granules could be due to degeneration and lack of contractility of myoepithelial cells around the acini and their inability to open into the lumen. The submandibular gland of 4w control group showed normal architecture of the glandular acini and the striated ducts (Fig 6C). The specimens of 4w diabetic group presented with large amount of stagnant secretory granules in some acinar cells and their lumen (Fig 6D) compared with 4w control group.

**Fig 7:**
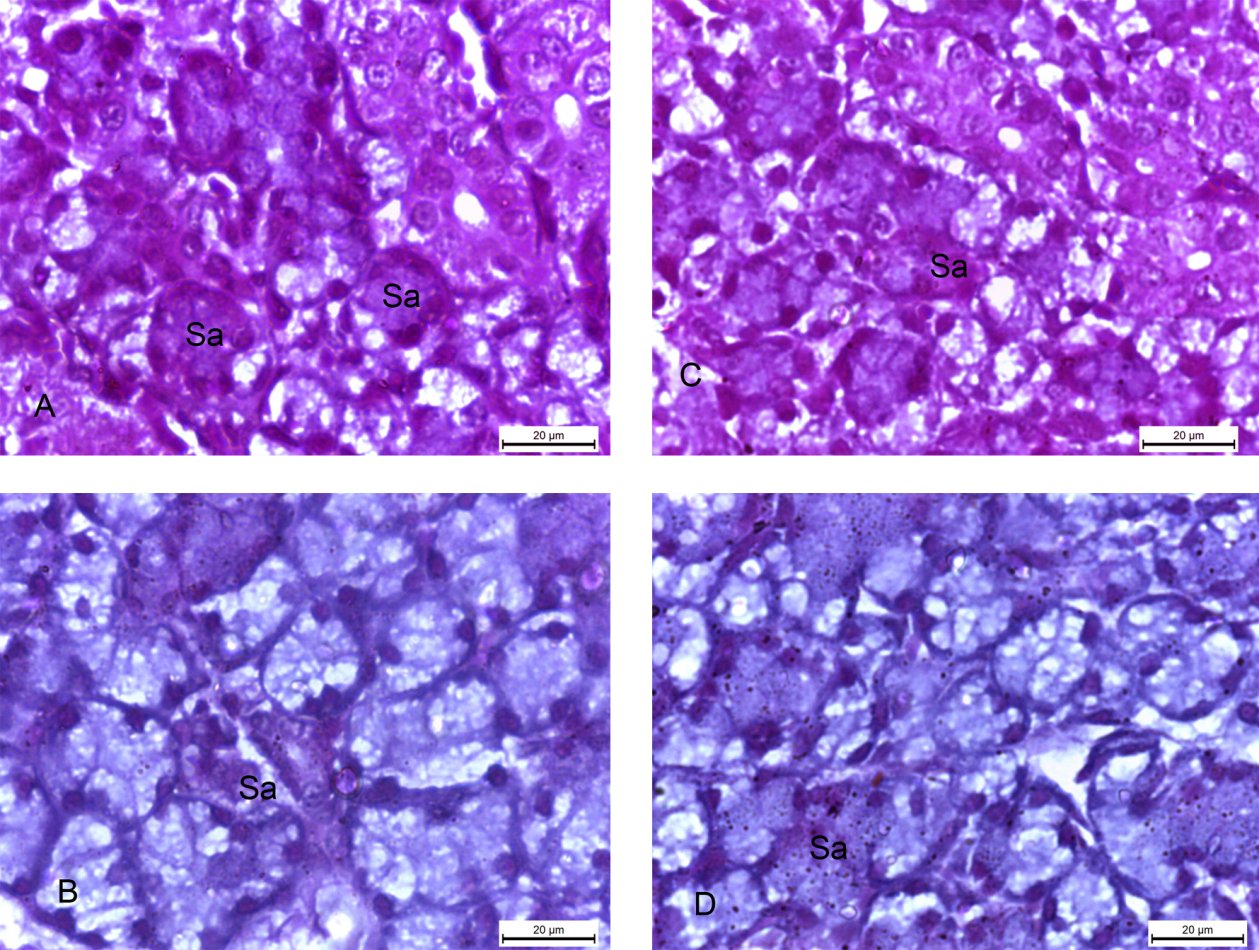
Photomicrographs of submandibular glands: A and C) (2w control and 4w control groups) showing strong positive magenta colour of PAS reaction of the cells of the serous acini (Sa). B and D) 2w diabetic and 4w diabetic groups) showing minimal positive PAS reaction in the serous acini (Sa). (PAS x 1000).

The submandibular glands of the 2w control and 4w control groups, stained with PAS stain, showed strong positive magenta colour of PAS reaction with the polysaccharides of the cells of the serous acini (Fig 7A and 7C), while the specimens of 2w diabetic and 4w diabetic groups showed minimal positive PAS reaction. (Fig 7B and 7D).

### The immunoreactivity of caspase-3, αSMA and PCNA

**Fig 8:**
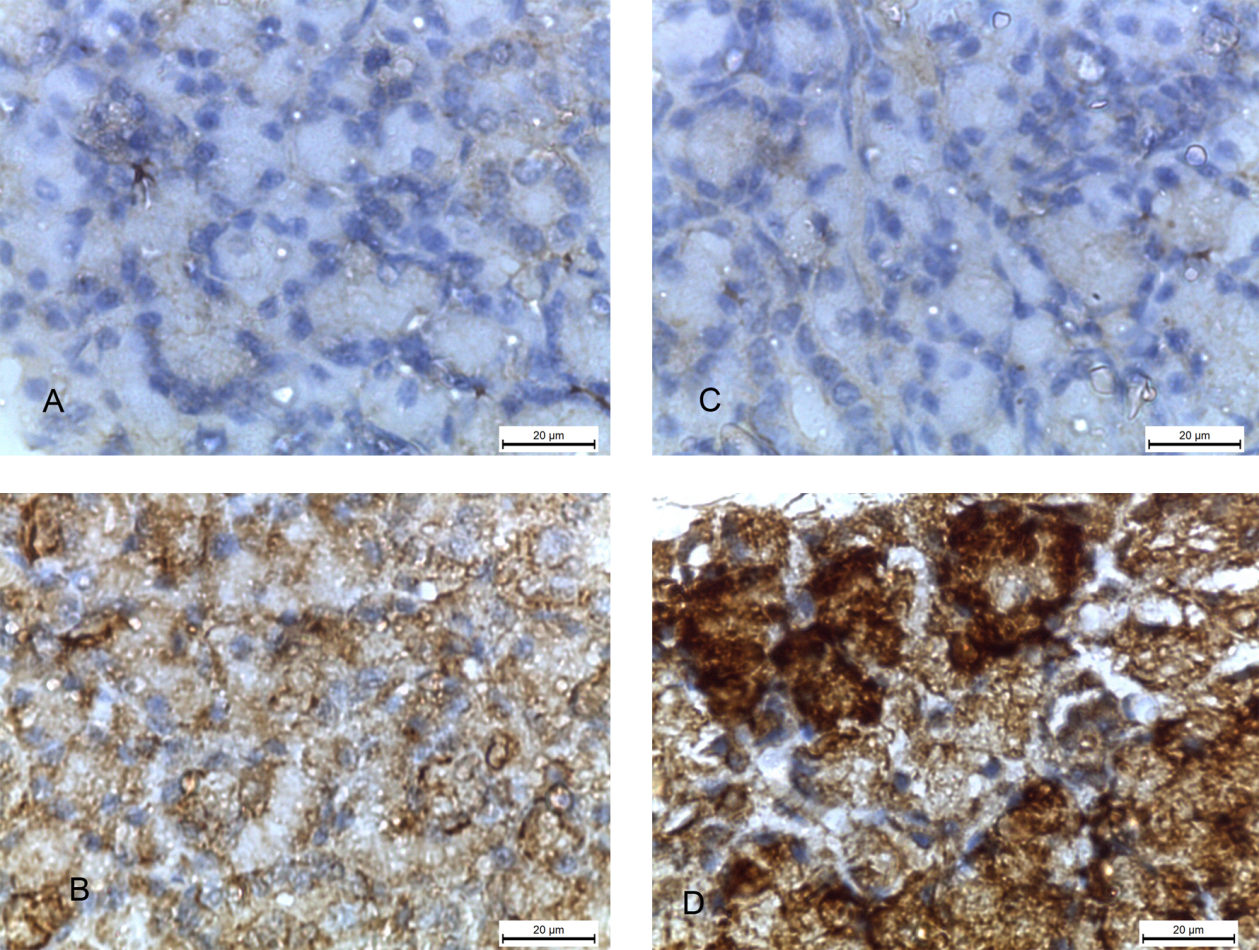
Photomicrographs of submandibular glands: A and C) (2w control and 4w control groups) showing negative caspase-3 reaction. B) (2w diabetic group) showing positive caspase-3 reaction in the form of brown discolouration of the cellular acini. D) (4w diabetic group) showing very strong positive caspase-3 reaction. (Caspase-3 x 1000).

The degree of apoptosis in the cellular cytoplasm was shown by immunohistochemical staining with caspase-3. The specimens of the 2w control and 4w control groups showed complete negative caspase-3 reaction (Fig 8A and 8C) while 2w diabetic group showed positive reaction in the form of brown discoloration of the cytoplasm of the acinar cells (Fig 8B). In addition, the specimens of 4w diabetic group presented very strong positive reaction (Fig 8D).

**Fig 9:**
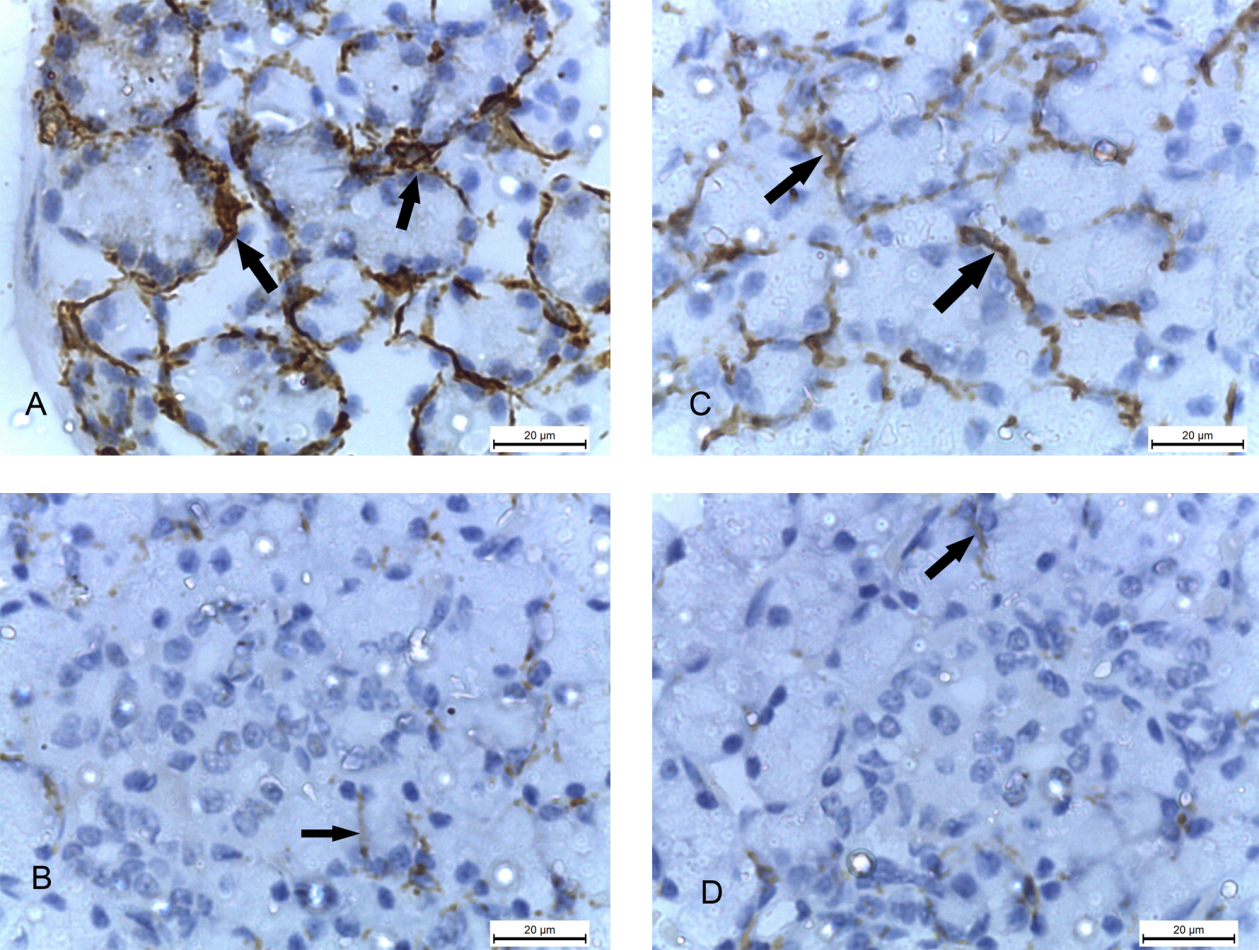
Photomicrographs of submandibular glands: A and C) (2w control and 4w control groups) showing strong positive α SMA reaction (arrows) around the acini. B and D) (2w diabetic and 4w diabetic groups) showing minimal α SMA reaction (arrows). (α SMA x 1000).

α SMA reaction showed the degree of myoepithelial cell proliferation, surrounding the glandular acini, in the form of brown deposits in their cytoplasm. The submandibular glands of 2w control and 4w control groups showed strong positive α SMA reaction around the acini (Fig 9A and 9C), while 2w diabetic and 4w diabetic groups showed minimal reaction (Fig 9B and 9D).

**Fig 10:**
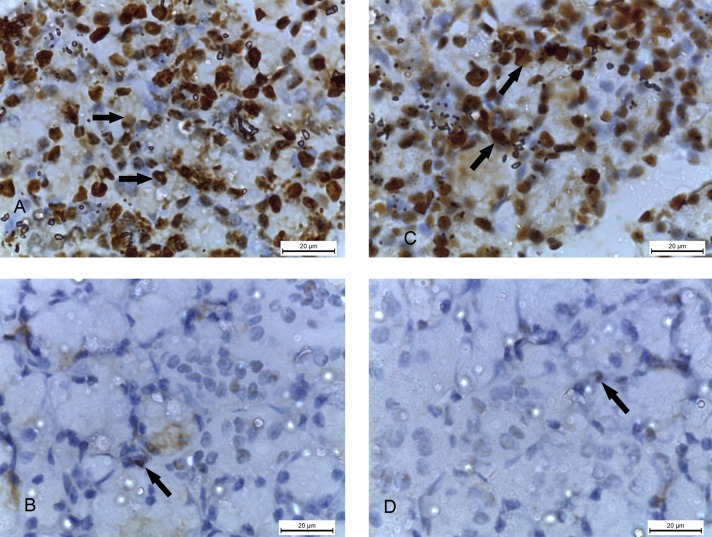
Photomicrographs of submandibular glands: A and C) (2w control and 4w control groups) showing strong positive PCNA reaction (arrows) in the cellular nuclei of the acini B and D) (2w diabetic and 4w diabetic groups) showing minimal PCNA reaction (arrows). (PCNA x 1000).

Immunohistochemical staining with PCNA showed the degree of proliferation in the nuclei of the cells of the acini and ductal epithelial cells. The specimens of the 2w control and 4w control groups showed strong positive PCNA reaction in the form of brown colored nuclei of the cellular acini (Fig 10A and 10C), while minimal reaction was found in 2w diabetic and 4w diabetic groups (Fig 10B and 10D).

**Fig 11:**
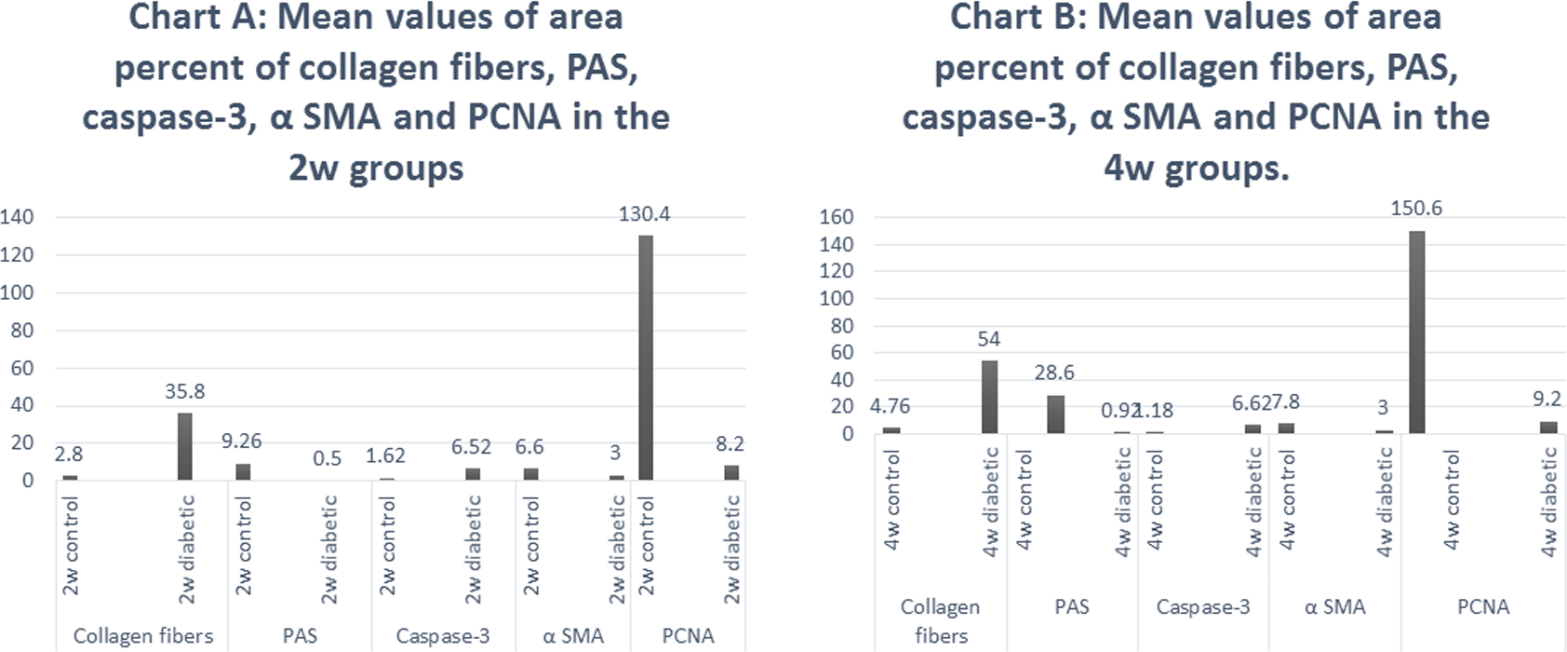
Chart A. Mean values of area percent of collagen fibers, PAS, caspase-3, α SMA and PCNA in the 2w control and 2w diabetic groups Chart B. Mean values of area percent of collagen fibers, PAS, caspase-3, α SMA and PCNA in the 4w control and 4w diabetic groups.

The area percent of collagen fibres and immune reaction for caspase-3 were significantly increased in 2w diabetic and 4w diabetic groups, compared with the 2w control and 4w control groups respectively. PAS reaction and immune reactions for α SMA and PCNA were significantly decreased in 2w diabetic and 4w diabetic groups, compared with the control groups (Tables 6 and 7 and Fig 11).

**Table 6:** Mean values, standard deviation and significance of area percent of collagen fibers, PAS, caspase-3, α SMA and PCNA in the 2w control and 2w diabetic groups.

	Case	Mean	Std. Deviation
Collagen fibers	2w control	2.80	1.48
	2w diabetic	35.80*	2.59
PAS	2w control	9.26	0.63
	2w diabetic	0.5*	0.16
Caspase-3	2w control	1.62	0.13
	2w diabetic	6.52*	0.38
α SMA	2w control	6.60	2.07
	2w diabetic	3.00*	1.58
PCNA	2w control	130.40	8.29
	2w diabetic	8.20*	1.30

*P value ≤ .05

**Table 7:** Mean values, standard deviation and significance of area percent of collagen fibers, PAS, caspase-3, α SMA and PCNA in the 4w control and 4w diabetic groups.

	Case	Mean	Std. Deviation
Collagen fibers	4w control	4.76	1.48
	4w diabetic	54.00*	3.39
PAS	4w control	28.60	2.30
	4w diabetic	0.92*	0.19
Caspase-3	4w control	1.18	0.24
	4w diabetic	6.62*	0.26
α SMA	4w control	7.80	1.30
	4w diabetic	3.00*	1.58
PCNA	4w control	150.60	3.85
	4w diabetic	9.20*	1.30

*P value ≤ .05

### Electron Microscopic Examination

**Fig 12:**
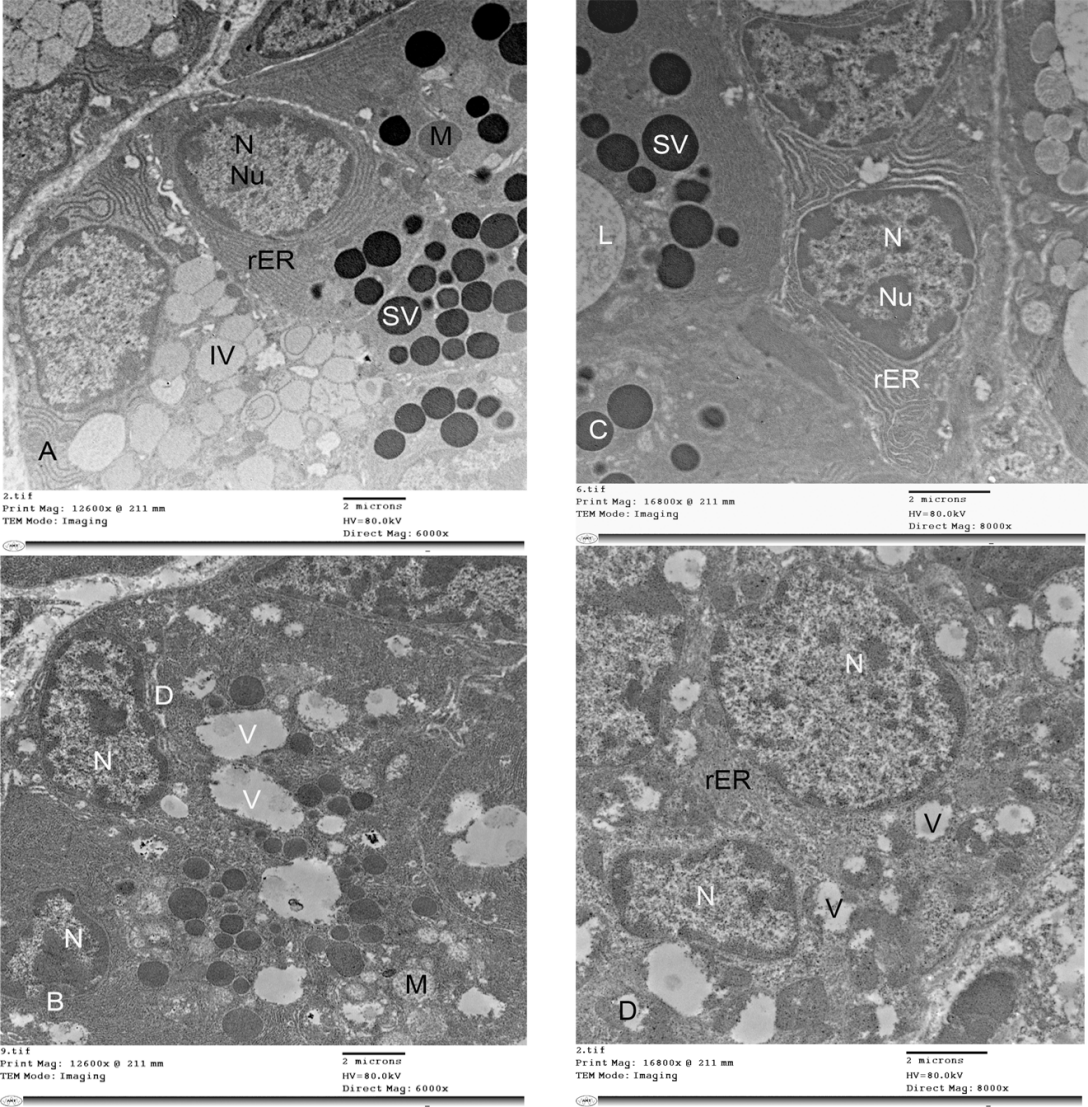
Electron photomicrographs of submandibular glands: A) (2w control group) showing homogenous variable sized mature secretory vesicles (SV) occupying most of the apical portion of the cytoplasm of the acinar pyramidal cells towards the lumen, immature vesicles (IV), rounded nucleus (N) and nucleolus (Nu), mitochondria (M) and parallel arrays of rough endoplasmic reticula (rER). B) (2w diabetic group) showing irregular dark nuclei (N), degenerated swollen mitochondria (M) with irregular cristae, irregular, dilated and degenerated rough endoplasmic reticula (D). The cytoplasm contains many large vacuoles (V) C) (4w control group) showing mature secretory vesicles (SV) in the cellular cytoplasm towards the lumen (L). Rounded nuclei (N) with nucleoli (Nu) and regularly packed rough endoplasmic reticula (rER). D) (4w diabetic group) showing irregular nuclei (N) with many cytoplasmic vacuoles (V) and irregular dilated degenerated rough endoplasmic reticula (rER). (A and B TEM x 6000, C and D TEM x 8000).

Specimens were taken from all the animals of the four groups to investigate the ultrastructure changes. The submandibular glands of 2w control group showed large pyramidal cells in the glandular acini. Homogenous variable sized mature secretory vesicles occupied most of the apical portion of the cellular cytoplasm towards the lumen. Immature vesicles appeared in some acini. Rounded nucleus and nucleolus, mitochondria and parallel arrays of rough endoplasmic reticula were seen in their cytoplasm (Fig 12A). The specimens of 2w diabetic group showed absence of mature secretory vesicles and the presence of only immature vesicles. The cytoplasm contained many vacuoles. The nuclei were pyknotic and irregular in shape. The mitochondria appeared degenerated and swollen with irregular cristae. Irregular dilated and degenerated rough endoplasmic reticula were scattered in the cytoplasm. The cytoplasm contained many large vacuoles and red blood cells (Fig 12B). These changes were detected in all the cells of the group with different level of pathogenicity. The specimens of 4w control group showed mature and immature secretory vesicles in the cellular cytoplasm towards the lumen. Rounded nuclei with nucleoli and regularly packed rough endoplasmic reticula were seen in the cytoplasm (Fig 12C). The glands of 4w diabetic group showed irregular nuclei with many cytoplasmic vacuoles. Large vacuoles were observed displacing and encroaching the shape of the nuclei. Irregular dilated degenerated rough endoplasmic reticula were scattered in the cytoplasm. Degenerated mitochondria were seen (Fig 12D). These changes were discovered in approximately all the cells of the group.

## Discussion

Loss of normal glandular architecture was found in the present study at the 2^nd^ and 4^th^ weeks old offspring of diabetic mothers. It was manifested by degenerative changes in the acini, atrophy in the striated duct and fibrosis around the ducts and in between the acini. These changes have been suggested to be due to nuclear and cytoplasmic apoptosis, cellular degeneration and failure of growth and development of the glands. Moreover, stagnation of secretory granules in the glandular acini found in the semithin sections could be due to lack of the contractility of myoepithelial cells surrounding the acini, demonstrated by negative reaction of α SMA, leading to accumulation of the secretion of the cells. The inability of these granules to open into the lumen might be due to alterations in the intracellular calcium (Ca^2+^) balance [16]. It was found that diabetes resulted in increase peroxidation of lipids with subsequent accumulation of Ca^2+^inside the cells, increase Ca^2+^ mobilizing ability of muscarinic receptors and reduction of Ca^2+^stores inside the endoplasmic reticulum. These changes of intracellular Ca^2+^were suggested to produce reduction of submandibular gland function [34]. These vesicles appeared in the electron microscopic study to be immature secretory vesicles. This criterion clarifies that maternal diabetes affected the submandibular gland functions. From these results, it could be suggested that intrauterine diabetic environment influences the development of the gland and its prenatal and postnatal structure and function. Reports, that studied the effects of diabetes on human submandibular salivary glands, recorded parenchymal atrophy and increase fibrous tissue that affect the gross morphology and produce sialadenosis [35]. Decrease in the number of secretory granules are in consistence with the belief that diabetes reduces the human gland secretory activity [36]. Another study attributed the impairment of rat salivary gland function to the decreased activity of Ca^2+^ -ATPases and increase Ca^2+^ influx with accumulation of Ca^2+^ in the mitochondria [34]. Cell swelling was also a consequence of electrolyte imbalance and changes in aquaporin distribution. Moreover, enlargement of secretory granules could be explained by changes in cell osmolarity and modified electrolyte concentration in the cytoplasm in rats [17]. In addition, diabetes was recorded to produce significant changes in human submandibular glands even with good glycemic control and absence of clinical signs of gland malfunction. The results were detected in ten patients with type two diabetes and mean age of sixty years indicating effects of diabetes on the submandibular glands. These changes were attributed to impaired glucose metabolism [16] and the relationship between intracellular glucose concentration and cell osmolarity producing cell swelling. Lasisi and Fasanmade [37] investigated the salivary flow rate and contents in forty adult human subjects. They found that diabetes significantly increased salivary glucose and potassium levels and reduced salivary flow rate. High salivary glucose level enhanced the possibility of oral infections, dental caries and improper wound healing. In addition, Ladgotra et al. [38], in their study on 120 adult human subjects, reported that diabetes increased glucose, calcium and phosphorus in saliva and reduced total salivary protein levels as result of hyperglycemia which directs protein utilization in other metabolic reactions. They recorded reduction of the level of salivary globulin, due to limited filtration of immunoglobulins down into the saliva compared with increase serum albumin leakage into the saliva, suggesting the possibility of low grade sialadenitis as a complication of diabetes.

The present study presented ultrastructural changes of the submandibular glands as a result of maternal diabetes. Degenerative changes in the nuclei, mitochondria and rough endoplasmic reticula were the main manifestations. These results are in agreement with previous studies, performed on rat models, attributed the cellular swelling of the serous acini to the impairment of glucose metabolism that affects cellular osmolarity producing electrolyte imbalance and changes in aquaporin distribution [17, 39]. Other studies investigated the glands of diabetic patients with controlled glycemia [15, 16]. The authors found significant enlargement of serous acini of diabetic patients. Degeneration of mitochondria was attributed as an effect of oxidative stress as a result of chronic hyperglycemia in diabetic patients [40, 41]. Oxidative damage, producing dysfunction of the rat salivary glands, could be the result of release of inflammatory cytokines (tumour necrosis factor (TNF), interleukin6 (IL6), and interleukin1β (IL1*β*)) which produce large amount of reactive oxygen species [42].

The significant reduction of PAS reaction, in the offspring of the of diabetic mothers, was attributed to decrease of polysaccharide concentration in the glandular acini. These results are consistent with other studies implemented on the rat submandibular glands [43, 44]. However, Nicolau et al. [45] found accumulation of glycogen in both parotid and submandibular glands of diabetic rats due to increase in glycogen synthase activity and reduction in the activity of glycogen phosphorylase.

Immunohistochemistry was performed in the current study to evaluate the degree of cellular apoptosis using caspase-3. Apoptosis, or programmed cell death with DNA fragmentation, is involved in cellular development and homeostasis. Multiple stimuli influence apoptosis, such as induction of diabetes that affects the regulation of submandibular gland structure which interprets the significant positive immune reaction to caspase-3 in the offspring of diabetic mothers and the diabetic rats themselves in the study of Maruo et al. [46]. Moreover, minimal reaction for α SMA, which is a myoepithelial cell proliferation marker [47], and PCNA, which denotes nuclear proliferation [48], in 2w diabetic and 4w diabetic groups demonstrates the degenerative effects of maternal diabetes on the glands of their offspring affecting their structure and function.

The current study quantitatively delineates, at the mRNA level, different markers in the submandibular gland of the offspring of diabetic mothers. Interestingly, our data demonstrated the degenerative effects of maternal diabetes on the development and growth of the submandibular salivary glands of the offspring presented by significant decrease in CK5, AQP5, EGF and Bcl2 in 2w diabetic and 4w diabetic groups. CK5 is a basal epithelial cell progenitor marker which produces both acinar and ductal submandibular gland epithelial cells [30]. CK5 expressing cells have been found to produce and reconstruct the epithelial cells of human submandibular glands and act as a potential diagnostic and therapeutic factors in tumours [49] suggesting that CK5 could have a diagnostic value for offspring tumorigenesis. This progenitor marker was suggested to contribute in gland development and homeostasis indicating that significant decrease of this factor found in the current study would markedly affect the morphology and fluid secretion of the submandibular glands. Expression of CK5 is linked with the response of salivary gland to cholinergic stimulation leading to secretion of saliva. Therefore, decrease in its expression results in xerostomia [32].

Moreover, AQP5, a water channel protein, is essential for allowing water to pass through the plasma membrane of mouse glands by osmosis [50, 51]. It has a significant functional role in saliva secretion of mouse submandibular gland [52]. It could act as an osmosensor due to its localization in the basal membrane of the acinar cells regulating paracellular fluid transfer [53]. Moreover, a strong association was detected between AQP5 and tight junction complexes that plays an important role in transcellular and paracellular fluid transport in the mouse parotid gland [52]. Therefore, significant decrease in AQP5 in the submandibular glands of the present study indicates the degenerative effect of maternal diabetes on the morphological differentiation and the secretory function of the glands. Maternal gestational diabetes may alter the normal oral flora of the offspring as it affects the salivary secretion [5]. Carpenter [3] reported that a decrease in the submandibular gland secretion results into loss of optimal oral pH needed for optimal growth of normal microbial oral flora. Moreover, diabetes results in deficiency of salivary amylase in the offspring causing a reduction in conversion of many insoluble complex polysaccharides into soluble simple unit that decreases the availability of substrates for the growth of oral microbes. Kaur et al. [54] reported that damage of salivary glands leads to hyposalivation and xerostomia that may cause stomatitis, dental caries due to decreased buffering capacity of saliva with overgrowth of the cariogenic bacteria, speech and chewing abnormalities due to loss of lubricant action of the saliva and abnormalities in taste sensation.

El Gamal et al. [29], Nelson et al. [31] and Takahashi et al. [32] recorded increase of these markers during normal rat and mouse submandibular salivary gland proliferation and development. EGF is secreted by the granular convoluted tubules. In diabetes, there is a decrease in protein synthesis that explains the weak expression of EGF in ductal cells [29]. Moreover, significant increase in Bax in the same groups indicates the glandular apoptosis which persisted to the 4^th^ week after birth. Expression of Bax could be responsible for cell destruction in mice [55] and programmed cell death in human tumours [56, 57]. The impacts and features of gene expression of these markers in the salivary glands of adult diabetic models and their offspring deserve further investigations.

## Conclusion

From the present work, it could be concluded that maternal diabetes produces degenerative effects in the structure and function of the submandibular salivary glands of the offspring, reflecting possible influences on their secretory activity affecting the oral and digestive health.
